# Perception of Knowledge Transfer from Clinical Simulations to the Care Practice in Nursing Students

**DOI:** 10.17533/udea.iee.v42n2e11

**Published:** 2024-07-08

**Authors:** Cristina García-Salido, Marina Mateu Capell, Daniel García Gutiérrez, Estel·la Ramírez-Baraldes

**Affiliations:** 1 Nurse. Ph.D. Professor. Email: cgarcia@umanresa.cat Universitat de VIC Spain cgarcia@umanresa.cat; 2 Nurse. Ph.D. Professor. Email: mmateu@umanresa.cat Universitat de VIC Spain mmateu@umanresa.cat; 4 Nurse. Ph.D. Professor. Email: eramirez@umanresa.cat Universitat de VIC Spain eramirez@umanresa.cat; 5 Department of Nursing, Faculty of Health Sciences at Manresa. Universitat de Vic- Universitat Central de Catalunya (UVic-UCC), Manresa (Barcelona); Spain. Universitat de VIC Department of Nursing Faculty of Health Sciences at Manresa Universitat de Vic- Universitat Central de Catalunya Manresa Barcelona Spain; 6 Intensive Care Unit. Althaia. Xarxa Assistencial Universitària de Manresa. Private Foundation. Manresa (Barcelona); Spain. Intensive Care Unit Althaia. Xarxa Assistencial Universitària de Manresa Private Foundation Manresa Barcelona Spain; 7 Research Group on Simulation and Transformative Innovation (GRIST), Instituto de Investigación e Innovación en Ciencias de la Vida y de la Salud de la Cataluña Central (Iris-CC), Ctra. De Roda Núm. 70, 08500 Vic, Spain. Universitat de VIC Spain

**Keywords:** patient simulation, simulation training, nursing, knowledge management, simulación de paciente, entrenamiento simulado, enfermería, gestión del conocimiento, simulação de paciente, treinamento por simulação, enfermagem, gestão do conhecimento

## Abstract

**Objective.:**

This work sought to assess the perception of knowledge transfer from clinical simulations to the care practice in nursing students through effective debriefing.

**Methods.:**

An observational, descriptive, and cross-sectional study was conducted with a sample of 281 students during the 2020-2021 course, through una *ad hoc* survey from the *Debriefing Assessment for Simulation in Healthcare* (DASH) in Spanish, to assess competence areas that undergraduate students must reach to complete their studies.

**Results.:**

The survey conducted after each simulation showed that the students valued positively the debriefing sessions conducted by experts, with a mean score of 6.61 over 7 [6.56%-6.65%] based on 675 surveys analyzed, given that each student conducted more than one simulation within the academic course. It was observed in 221 completed answers that what was learned in the simulation was transferred to the practice in 89.23% [86.39%-92.06%], specifically in areas of Communication, Patient safety, Teamwork, and Leadership.

**Conclusion.:**

In the perception by the participating students, the use of effective debriefing in clinical simulation enabled knowledge transfer to the care practice, proving to be a crucial tool that helps to improve the formation of the future nurses.

## Resumen

**Objetivo.:**

Evaluar la percepción de la transferencia de conocimientos de las simulaciones clínicas a la práctica asistencial en estudiantes de enfermería mediante un *debriefing* eficaz.

**Métodos.:**

Se realizó un estudio observacional, descriptivo y transversal con una muestra de 281 estudiantes durante el curso 2020-2021, mediante una encuesta *ad hoc* a partir de la *Debriefing Assessment for Simulation in Healthcare* (DASH) en español, para valorar las áreas competenciales que un estudiante de grado debe alcanzar al finalizar sus estudios.

**Resultados.:**

La encuesta realizada después de cada simulación mostró que los estudiantes valoraban positivamente las sesiones de *debriefing* realizadas por expertos, con una puntuación media de 6.61 sobre 7 [6.56 % - 6.65 %] en base a 675 encuestas analizadas, ya que cada estudiante realizaba más de una simulación dentro del curso académico. Se observó en 221 respuestas cumplimentadas que lo aprendido en la simulación se transfirió a la práctica en el 89.23 % [86.39 % - 92.06 %], específicamente en áreas de Comunicación, Seguridad del paciente, Trabajo en equipo y Liderazgo.

**Conclusión.:**

En la percepción de los estudiantes participantes, el uso de un *debriefing* eficaz en la simulación clínica permitió la transferencia de conocimientos a la práctica asistencial, mostrando ser una herramienta crucial que ayuda a mejorar la formación a de los futuros enfermeros.

## Resumo

**Objetivo.:**

Avaliar a percepção da transferência de conhecimentos das simulações clínicas para a prática assistencial em estudantes de enfermagem por meio de um *debriefing* eficaz.

**Métodos.:**

Foi realizado um estudo observacional, descritivo e transversal com uma lista de 281 estudantes de enfermagem de uma universidade espanhola durante o curso 2020-2021, por meio de uma consulta ad hoc a partir do *Debriefing Assessment for Simulation in Healthcare* (DASH) em português, para valorizar as áreas competentes que um estudante de graduação deve alcançar ao finalizar seus estudos.

**Resultados.:**

A consulta realizada após cada simulação mostrou que os estudantes valorizaram positivamente as sessões de *debriefing* realizadas por especialistas, com uma pontuação média de 6.61 sobre 7 [6.56-6.65%] com base em 675 consultas analisadas, e cada estudante realizou mais de uma simulação dentro do curso acadêmico. Foram observadas 221 respostas cumulativas que o aprendizado na simulação se traduziu na prática em 89.23% [86.39-92.06%], especificamente nas áreas de Comunicação, Segurança do paciente, Trabalho em equipe e Liderança.

**Conclusão.:**

Na percepção dos estudantes participantes, o uso de um *debriefing* eficaz na simulação clínica permitiu a transferência de conhecimentos para a prática assistencial, mostrando ser uma ferramenta crucial que ajuda a melhorar a formação dos futuros enfermeiros.

## Introduction

Learning in the Spanish health education system, established in 1977, focuses on acquiring necessary abilities and skills for the nursing care practice nurse, guaranteeing patient safety and quality of care.^1^ With the introduction of new health teaching technologies based on competence areas in the European Higher Education Area since the year 2000, it became necessary to incorporate these advances to the nursing curriculum.^(2)^ Clinical simulation has emerged as a valuable educational tool in diverse fields, with the aim of improving the professional practice, minimizing errors, and promoting professionals towards excellence. Due to its use, it is possible to recreate scenarios that resemble real patient care situations where students face the challenge of applying their abilities, knowledge, and clinical attitudes, thus integrating theory and practice. This methodology usually consists of three main phases: predebriefing, simulation and debriefing.^3^ The debriefing, a crucial component of the experimental learning process that encompasses reflection, feedback, and future experimentation, allows students to develop and integrate knowledge acquired during direct experience.^4^^,^^5^

Simulation scenarios are classified in different zones (from 0 to 4) in function of their complexity, starting with learning clinical abilities and technical procedures (zone 0 and 1) and advancing to teamwork training in crisis situations and human factors (zona 2 a 4).^6^ In 2014, the Degree in Nursing from the Faculty of Health Sciences at Manresa of Universitat de Vic- Universitat Central de Catalunya (UVic-UCC) incorporated the clinical simulation as key methodology to prevent errors, improve care practices, and guarantee patient safety. The clinical simulation sessions were aligned with specific themes of the study plan to educate nursing students about providing professional care, acquisition of legal and ethical standards, emphasizing on safety and quality.

To assess the effectiveness of the clinical simulation, two scales have been used internationally: the *Debriefing Assessment for Simulation in Healthcare* (DASH), developed by the Boston Center for Medical Simulation, centered on evaluating the debriefing process from the perspectives by students, evaluators, and instructors;^7^ and the Satisfaction with Simulated Clinical Experiences scale, validated in Spanish by Negrão *et al*.,^8^ and which measures satisfaction by nursing students with simulated clinical sessions in three dimensions: practice, realism, and cognitive. 

Although these scales provide information about perceptions and student satisfaction, they do not evaluate specifically the transferability of the competences practiced in the clinical simulation to the real care practice. Previous studies^9^ indicate that simulations represent an advantage for health workers as they progress in reflective learning, clinical judgment, and decision making. Additionally, simulation scenarios represent a close, safe and non-punitive environment, where students can practice techniques in controlled, supervised manner, with the possibility of errors and unlimited repetition.^10^ Currently, studies exist^11^ that assess student satisfaction in relation to their participation in the simulation. Another study^12^ adds the debriefing evaluation, as previously discussed with the DASH, but no scale was found in the literature that measures knowledge transfer from clinical simulation to care practices, being a challenge to be achieved by the community of professors who teach or practice this simulation methodology. The aim of this research was to evaluate the perception of knowledge transfer from clinical simulations to the care practice in nursing students through effective debriefing.

## Methods

With the purpose of contributing to understanding how an effective or good quality debriefing in clinical simulation facilitates knowledge transfer to the care practice and improves the formation of future nurses.

Population and sample. The entire study population included 428 nursing students registered in the Nursing Degree of the Faculty of Health Sciences at Manresa of Universitat de Vic- Universitat Central de Catalunya (UVic-UCC) with a final sample of 281 participants. The study sample was determined through convenience and included all the students who had participated in simulations and clinical practices during the academic year 2020-2021. 

Type of study with observational, descriptive, and cross-sectional quantitative methodology.

### Techniques and procedures

Two surveys were used: “1^st^ Post Clinical Simulation Survey” and “2^nd^ Post-Practicum Survey” to analyze the students' perception of their knowledge transfer from clinical simulations to care practice.

Before starting the study, the researchers obtained approval from the Ethics Committee at Universitat de Vic- Universitat Central de Catalunya (UVic-UCC), students were informed of the study objective, of their voluntary and anonymous participation, after signing the informed consent prior to starting the simulations that include using recorded material for educational and/or research purpose. To achieve effectiveness and/or quality of the debriefings, it was guaranteed that all simulation instructors had a postgraduate or master's degree in simulation. 

The clinical simulation sessions were classified into zones 2 and 3, centered on fostering teamwork, the practice of systematic processes integrated in clinical cases and development of the capacity to make decisions. First-course students performed a simulation about communication skills; those from the second course carried out four simulations of cardiology, home care, nutrition and therapeutic communication; the third-course students conducted three simulations about hematology, healthy-child evaluation and immediate life support, and finally, those from the fourth course performed three simulations in pediatric advanced life support, adult advanced life support and complex chronic patient (CCP). 

The first questionnaire, the "1^st^ Post Clinical Simulation Survey", was elaborated from the Debriefing Assessment for Simulation in Healthcare (DASH) in Spanish, specifically the extended version for students,^13^ adding questions related to gender, course, academic year, practicum and simulation case. The DASH in Spanish was chosen because of its effectiveness and capacity to gather the students’ perspectives about the quality of the debriefing by their instructors, assessing the behaviors by the professors who facilitate learning and change in experimental settings through the analysis and assessment of six elements: Element 1 - The instructor established an environment for a participative learning experience; Element 2 - The instructor maintained an environment of participative learning; Element 3 - The instructor structured the debriefing in organized manner; Element 4 - The instructor generated profound discussions that made me reflect about my performance; Element 5 - The instructor identified what I did well or poorly; and Element 6 - The instructor helped me to see how to achieve or maintain good performance. 

Upon completing the simulation, using a link in a QR code, students assessed each of the elements, with a Likert scale from 1 to 7, thus: 1 = Extremely ineffective / Harmful; 2 = Consistently ineffective/Very poor; 3 = Mostly ineffective/Poor; 4 = Somewhat effective / Average; 5 = Mostly effective / Good; 6 = Consistently effective / Very good; and 7 = Extremely effective / Outstanding. For such, prior permission was requested from the authors of the DASH in Spanish for its use and digitization on the Redcap platform. 

The "2^nd^ Post-Practicum Survey", of *ad hoc* elaboration*,* was created bearing in mind the competence, cross-sectional, and specific areas nursing students must achieve upon ending the at the end of the course, which was agreed upon through the Delphi method, with participation by the emerging group recognized by the Generalitat of Catalonia for Research in Teaching Innovation, Simulation, and Patient safety, along with the teaching staff from the Nursing Program of the Faculty of Health Sciences at Manresa in Universitat de Vic- Universitat Central de Catalunya (UVic-UCC). This second survey was also digitized and made accessible through the Redcap platform. This survey sought to know students’ perception about the transfer of knowledge acquired during the simulations to the care practices upon completing their practical formation and return to the university. 

The "2nd Post-Practicum Survey" is divided into two sections: a *first part* corresponding to the transversal competence areas worked on throughout each of the simulations of the nursing degree (communication and patient safety as of the first course, teamwork as of the third course, and leadership only during the last year), where the participating students respond to their perception about the transference from the simulation to their clinical practices, more generally, through questions related with their activities during their practice period. A *second part* in which specific competence worked in concrete simulation cases (basic communication skills, cardiology, home care, nutrition, therapeutic communication, hematology, healthy child evaluation, immediate life support, pediatric advanced life support, adult advanced life support, and chronic complex patient) to observe if that learnt during simulation had been applied in their places of practice, through questions based on their competence level. 

Data were analyzed with the STATA, v. 17 statistical program. Each variable was described by calculating means and 95% confidence intervals, with 5% margin of error. Each demographic variable was described globally by course and specifically for each simulation, as well as the competences acquired in the simulations and transferred to the care practices. Regarding the "1^st^ Post Clinical Simulation Survey”, each student answered it out after each simulation case, bearing in mind the teaching program described above where more than one session is carried out in each course. Due to this, from the 281 students participating, 675 surveys were analyzed, of which 93.63% were female. By academic year, they were distributed thus: 99 (14.67%) in the first; 309 (45.77%) in the second; 149 (22.07%) in the third; and 118 (17.48%) in the fourth. 

## Results

Throughout the four courses and for all the simulations, the mean global score obtained for the six elements of the DASH in Spanish was 6.61 over 7.00 [95% CI: 6.56-6.65] (Table 1). 

**Table 1:** Mean global score of the DASH in Spanish according to course, simulation, and element

Course	Simulation	Mean	Error	95CI of the mean
First (*n*=99)	Communication skills	6.51	0.08	6.36-6.66
Second (*n*=309)	Cardiology	6.58	0.09	6.39-6.76
	Home care	6.62	0.07	6.49-6.67
	Nutrition	6.8	0.06	6.68-6.93
	Therapeutic communication	6.77	0.03	6.70-6.84
Third (*n*=149)	Hematology	6.62	0.05	6.51-6.72
	Healthy child evaluation	6.54	0.09	6.37-6.71
	Immediate life support	6.49	0.31	5.88-7.00
Fourth (*n*=118)	Pediatric advanced life support	6.56	0.17	6.23-6.89
	Adult advanced life support	6.79	0.15	6.49-7.00
	Chronic complex patient	6.43	0.08	6.26-6.60
Evaluation elements (*n*=675)				
E1: The instructor established an environment for participative learning experiences		6.47	0.16	6.31-6.62
E2: The instructor maintained an environment of participative learning		6.64	0.165	6.51-6.84
E3: The instructor structured the debriefing in organized manner		6.63	0.08	6.56-6.71
E4: The instructor generated profound discussions that made me reflect about my performance		6.63	0.02	6.59-6.63
E5: The instructor identified what I did well or poorly - and why		6.63	0.14	6.51-6.78
E6: The instructor helped me to see how to achieve or maintain good performance		6.63	0.09	6.48-6.65
Total		6.61	0.05	6.56-6.65

Of the 281 participants, 221 (representing 78.64%) had the opportunity during the practice period of experiencing the same cases as those worked on in the simulation; 83.20% were females and, by course, they were distributed thus: 45 students from the first, 84 from the second, 61 from the third, and 31 from the fourth. Regarding the transversal competence areas worked on during the simulations, the areas of *patient safety*, globally, 81.00% [78.77%-83.28%] of the students agreed that what was learnt during the simulation was transferred to the practice. The most transferred aspects were use of gloves with 99.52% [98.66%-100%] and introduction with name and professional category with 95.91% [93.30%-98.55%] for the four courses. Nevertheless, second-course students were the ones who least considered that this knowledge was transferred with 75.40% [71.88%-78.95%]; specifically, the least transferable aspects were: use of railings with bedridden patients with 54.82% [43.99%-65.53%], bringing the bell closer to a dependent patient with 69.00% [59.05-79.05], and verification of allergies with 40.53% [29.86%-51.09%]. Moreover, overall, the students reported that the simulations helped them to integrate strategies to improve this competence area with 78.50% [71.68%-85.27]. 

Additionally, the *communication* competence was the most transferred to the clinical practice with a mean of 95.40% [94.31%-96.46%] in all courses, with the first course having the highest rate with 97.30% [95.63%-99.04%] and the third course having a rather low rate with 94.10% [92.07%-96.13%]. The most-transferred item of communication was active listening, with 100% incidence and, on the contrary, the least-transferred item, although high at global rate, was effective communication among team members with 80.60% [66.43%-94.86%]; finally, the students stated that the simulations helped them to integrate strategies to improve this competence with 81.90% [76.78%-87.02%]. 

At the same time, teamwork, a competence of the third and fourth courses, obtained an overall transferability of 92.20% [89.57%-94.78%]; while the most-transferred aspect was respect for the members of the care team with 100%. It is fitting to note that the fourth-course students expressed 100% transferability in the integration of the care staff, communication among the care team, and recognition of teamwork in solving specific situations. Rather, in the third course the least valued items were communication and non-integration in the care team during the practice period, both with 96.70% [92.15%-100%]. Additionally, they stated that the simulations helped them to integrate strategies to improve this competence area by 60.70% [48.13%-73.18%]. 

Lastly, the *leadership* competence was only assessed in the fourth course, given that it is a competence area that is only worked on in said course, thereby, there was no comparison with previous courses. Said competence showed transference of 88.30% [82.89%-93.72%] of the knowledge learnt; the items with the highest scores were those related to the students' initiative to identify and solve situations and problems related with the staff or with the patients, with 100%, instead, the item with the lowest score was that related with not identifying the leader figure and not identifying orders with 87.13% [74.60%-99.60%]. Finally, the students reported that the simulations helped them to integrate recognition of the leader figure in 71.00% [54.04%-87.89%] and obtain the necessary knowledge to follow their orders in 67.77% [50.31%-85.17%]; for further information, see Table 2.

**Table 2:** Global transferability rate per hundred of the transversal competence areas worked on in the simulations

Course / area			Patient safety	Communication		Teamwork	Leadership
First (*n*=45)	%	85.20	97.30	.	.
	95% CI	80.72-89.68	95.63-99.04	.	.
Second (*n*=84)	%	75.40	95.50	.	.
	95% CI	71.88-78.95	93.52-97.43	.	.
Third (*n*=61)	%	83.60	94.10	90.50	.
	95% CI	79.09-88.12	92.07-96.13	86.94-94.04	.
Fourth (*n*=31)	%	85.10	94.80	95.50	88.30
	95% CI	79.56-90.58	91.97-97.71	92.45-98.52	82.89-93.72
Total (*n*=221)	N	221	221	92	31
	%	81.00	95.40	92.20	88.30
95% CI	78.77-83.28	94.31-96.46	89.57-94.78	82.89-93.72

Reference is made to the second section of the survey "2^nd^ Post-Practicum Survey" of the specific competence areas worked on in the simulations. The first-course students manifested a transfer rate in basic communication skills of 83.70% [73.37%-94.07%], with the simulation related with active listening having the highest transference value of 86.00% [75.26%-96.84%] and the lowest related with direct communication with different types of patients with care purposes, uncooperative or collaborative or nervous and with their family environment of 81.40% [69.28%-93.51%]. The second course expressed a transfer rate in the management of the nursing consultation in nutritional education of 85.50% [76.47%-94.50%], as for the highest value of 98.50% [95.46%-100%] in validating the patient's understanding of their nutritional plan and the lowest value of 85.50% [76.47%-94.50%] in offering appropriate dietary recommendations. 

During the motivational interview, the students reported a transference of 80.00% [70.01%-89.99%], with the highest value in the application of the patient’s emotional containment, as well as in techniques of emotional support and active listening with 98.50% [95.59%-100%] and the lowest value of 80.00% [70.01%-89.99%] in providing reinforcement and motivation for adherence to treatment. With respect to nursing care in cardiology care, there was a transfer rate of 69.33% [57.96%-80.73%], with the highest value of 93.30% [79.03%-100%] related with performing an electrocardiogram, and the lowest, of 68.20% [47.04%-89.32%] in the application of postoperative knowledge in pacemaker surgical interventions. Finally, in home nursing care they reported a transfer rate of 83.10% [73.71%-92.44%], with the highest value, of 91.10% [83.37%-98.78%] in relation with the nursing diagnosis, and the lowest value in relation with the registry of patient care and needs, with 82.50% [72.90%-92.18%]. The third course expressed a transfer rate of 66.70% [50.49%-82.84%] in nursing care with hematological alterations, regarding extraction of blood samples, checking the blood groups, and performing blood transfusions. In immediate life support of 66.70% [28.23%-100%] conducting the ABCDE nursing assessment and in pediatric nursing care of 69.40% [56.01%-82.76%], with recommendations for breastfeeding, vaccination schedule, and administration of usual medication. The fourth course manifested a transfer rate in nursing care of chronic complex patients in primary care of 82.60% [65.85%-99.37%] with the performance of the nursing assessment and application of tests or functional scales. Regarding adult advanced life support of 80.00% [60.79%-99.21%] and pediatric life support of 100%, tracking algorithms, monitoring and identifying cardiac rhythms and administering medication during cardiorespiratory arrest. Finally, for the advanced practice nurse it was 75.00% [46.26%-100%] with the implementation of recommendations and follow-up guidelines. 

Lastly, globally, students who were able to carry out simulation cases and apply them in care practices, reported that the simulations helped 76.66% [63.89%-89.59%]: with this being higher in the fourth course (85.90%) and lower in the third course (67.60%); they were also able to transfer knowledge from said simulations to their role as professional nurses in 81.70% [72.08%-91.90%]: being higher in the fourth course (95.60%) and lower in the third course (77.00%) (Table 3).

**Table 3:** Global transferability rate per hundred of the specific competence areas worked on in the simulations per course

Course / Question	*n*	%	95% CI
Have the simulations helped you?			
First	.	.	.
Second	78	79.60	72.81-86.49
Third	55	67.60	44.91-88.53
Fourth	27	85.90	70.70-100
Total	160	76.66	63.89- 89.59
Have you been able to apply knowledge / skills?			
Course	*N*	%	95% CI
First	43	83.70	73.37-94.07
Second	79	79.60	79.51-92.11
Third	55	77.00	85.21-93.34
Fourth	27	95.60	90.69-100
Total	204	81.70	72.08-91.9

## Discussion

The findings herein regarding the effectiveness of the debriefing in the clinical simulation, according to the results obtained from the DASH, indicate a positive perception by the students. These results suggest that the instructors have the capacity to create a suitable environment for reflexive learning through organized sessions that promote profound discussions about the student’s performance. Previous research in nursing programs have yielded results similar to the present project with 6.61 [6.56-6.65], ranging between 6 and 6.50; highlighting the need to improve the introduction at the beginning of the simulation activities (element 1), as well as the instructor’s ability to maintain a participative learning environment (element 2).^14^^-^^19^ Other studies focused on the formation of health professionals have also obtained similar assessments as the present study, suggesting that the debriefing was effective and of high quality^20^. The importance is underscored of maintaining a safe environment and promoting professional integrity during the simulation, recognizing its profound impact on health students.^21^ Additionally, the crucial relevance of the experience and training of professors in clinical simulation to guarantee student satisfaction is highlighted.

With respect to knowledge transfer to the clinical practice, most students state that knowledge acquired during the clinical simulations applies effectively in the care practice. The data reveal that 81.00% of the students consider that what they learnt during the simulations on patient safety was transferred to the practice, with gloves and adequate introduction with name and professional category obtaining transferability rates close to 100%. Furthermore, it is highlighted that 95.40% of the students experienced high transference of communication skills, with active listening being a 100% universally transferred element. In teamwork, it is observed that 92.20% of the students experienced effective transference of abilities, highlighting respect for staff members as a 100% universally transferred aspect. 

These findings coincide with prior scientific evidence^22^ that support the effectiveness of the clinical simulations in the transference of skills to the clinical practice. Previous research ^23^^,^^24^ have demonstrated that clinical simulations offer a safe and controlled environment where students can practice and consolidate clinical skills before facing real situations with patients. In this sense, simulations not only serve as effective teaching tools, but also contribute significantly to the development of competencies crucial for quality care in real clinical settings.

Although different programs exist ^25^^,^^26^ in nursing based on simulation, this study provides the novelty of determining the differences in the perception of knowledge transfer and competence areas among the courses of the university nursing degree. For example, the data show that students from the first course have a high transferability rate in basic communication skills, such as active listening and empathy, which suggests effective Integration of these skills as of early training stages. This finding may be supported with prior studies^27^ that highlight the importance of developing effective communication skills during the first years of nursing formation to establish a solid base for the clinical practice. In turn, the perception of lower transferability in aspects of patient safety by the students from the second course is also supported in the literature. Previous research^28^ have identified deficiencies in knowledge and the application of patient safety protocols among nursing students during intermediate stages of their training. This suggests the need for specific educational interventions, like the Integration of simulation scenarios focused on patient safety, to address these critical areas of the clinical practice and improve the perception of transferability of knowledge in this group of students.

These data reinforce the importance of understanding the areas of greater and lesser knowledge transfer in the nursing formation.^22^ According to the results presented, the areas with greater transference include the use of gloves, introduction with name and professional category, active listening, and respect toward the members of the healthcare staff. In contrast, the areas with lesser knowledge transfer include, among others, the use of railings with bedridden patients, bringing the bell closer to a dependent patient, and verification of allergies in students from the second course. It was found that communication and integration in the healthcare staff during the practices, and identification of the leader figure and the leader’s orders had lower transferability rates in students from the third and fourth courses, respectively. 

Hence, adapting the clinical simulations to the specific needs of each cohort of students and competence area is justified not only due to the differences observed in the perception of transferability, but also by prior evidence^22^ that supports the importance of addressing these specific areas of professional development during the nursing formation. These personalized strategies have the potential of both improving student confidence and competence, and can have a positive impact on the quality of patient care in real clinical settings.^21^

The study acknowledges certain limitations, such as the use of convenience sampling and dependence on the subjective perception of students. Future research should incorporate mixed methods to obtain fuller comprehension of how the debriefing in the clinical simulation impacts upon the care practice. In addition, strategies can be explored to improve knowledge transfer in areas where lower transferability rates are observed.

For decades, nursing literature has indicated the existence of a gap ^29^ between academic theory and practical application, with limited evidence of significant progress to reduce this disparity and this research emphasizes the simulation’s potential as an educational strategy to approach this gap, given that the results show that this educational methodology is effective to increase the students’ perception in their transition to clinical care practice. This project offers results that confirm the scientific evidence ^30^^-^^32^ that simulation of the clinical practice has transference of the learning by the nursing students, contributing to their knowledge, abilities, confidence, and judgement. Nonetheless, the study agrees with other studies^24^^,^^29^ on the need to continue exploring this setting and the need is identified for more longitudinal research to further delve into the long-term learning results from simulation. 

The debriefing, as a reflection and analysis process after the clinical simulation experiences, is universally recognized as an essential component to clarify and consolidate the knowledge acquired during these practices. The results of the DASH-based "1^st^ Post Clinical Simulation Survey" provide evidence that the professors who facilitated the clinical simulation exhibited the necessary behavior to conduct an effective debriefing with our nursing students. Moreover, the "2^nd^ Post-Practicum Survey" revealed that the students perceive that the clinical simulation allows them to transfer knowledge from transversal competence areas to the care practice, enhancing their capacity in patient safety, therapeutic communication, teamwork, and leadership. In this sense, the research offers a significant contribution to the field of nursing education upon identifying specific areas of greater and lesser knowledge transfer among students from different courses and competence areas. The findings establish that, although there is a solid integration of basic communication skills, such as active listening and empathy, from the initial stages of training, deficiencies persist in critical clinical practice areas, like patient safety and leadership in healthcare teams.

These results support continuing and strengthening learning based on clinical simulation for nursing students, given that this methodology provides an effective platform to apply essential concepts and abilities, thus, improving the quality of patient care and attention. Furthermore, these findings suggest that the simulations in the classroom help nursing students to close the gap between acquiring knowledge and applying them to patient care.

In conclusion, this study provides conclusive evidence about the effectiveness of debriefing in clinical simulation to facilitate knowledge transfer to the care practice among nursing students. The results emphasize the importance of a structured and reflexive debriefing to enhance clinical competence and improve patient care in future nursing professionals. Likewise, these back the need to adapt clinical simulations to the particularities of each student group and competence area. This adaptation is sustained not only on the discrepancies identified in the perception of transferability, but on previous evidence that underscores the relevance of addressing specific areas of professional development during the nursing formation. These tailored strategies can increase student confidence and competence, generating a positive impact on the quality of patient care in real clinical settings. 

To end, future research is considered necessary to address variations between educational programs and competence areas, as well as to continue optimizing the design and application of clinical simulations in nursing formation to guarantee its effectiveness and relevance within the context of the practice professional. These types of research could delve into identifying the best practices in debriefing and in the adaptation of simulations to the specific needs of each student cohort, which could contribute significantly to continuous improvement of formation in nursing and, lastly, to the offer of high-quality care to patients.

## References

[1] Arroyo Gordo MP (2009). La titulación de grado en enfermería y las competencias profesionales. Revista de Administracion Sanitaria.

[2] Gutiérrez I de la H (2010). La simulación clínica como herramienta de evaluación de competencias en la formación de enfermería. REDUCA (Recursos Educativos Enfermería, Fisioterapia y Podología).

[3] Akhtar-Danesh N, Baxter P, Valaitis RK, Stanyon W, Sproul S (2009). Nurse faculty perceptions of simulation use in nursing education. Western Journal of Nursing Research.

[4] Maestre JM, Szyld D, del Moral I, Ortiz G, Rudolph JW (2014). The making of expert clinicians: Reflective practice. Revista Clínica Española (English Edition).

[5] Rudolph JW, Simon R, Dufresne RL, Raemer DB (2006). There’s No Such Thing as “Nonjudgmental” Debriefing: A Theory and Method for Debriefing with Good Judgment. Simulation in Healthcare. The Journal of the Society for Simulation in Healthcare.

[6] Roussin CJ, Weinstock P (2017). SimZones: An Organizational Innovation for Simulation Programs and Centers. Academic Medicine.

[7] Simon R, Raemer D, Rudolph J (2010). Debriefing Assessment for Simulation in Healthcare (DASH)© Rater’s Handbook.

[8] Negrão Baptist RC, Amado Martins JC, Ribeiro Pereira MFC, Mazzo A (2014). Satisfacción de los estudiantes con las experiencias clínicas simuladas: validación de escala de evaluación. Rev Latino-Am Enfermagem.

[9] Klenke-Borgmann L, Cantrell MA, Mariani B (2021). Clinical Judgment in Nursing Students After Observation of In-Class Simulations. Clinical Simulation in Nursing.

[10] Casal Angulo M del C (2016). La simulación como metodología para el aprendizaje de habilidades no técnicas en Enfermería. [Tesis Doctoral]. [Internet]. Programa De Doctorado En Enfermería.

[11] Juguera Rodríguez L, Agea Díaz, Luis J, Pérez Lapuente ML, Leal Costa C, Rojo Rojo A, Echevarría Pérez P (2014). La simulación clínica como herramienta pedagógica. Percepción de los alumnos de Grado en Enfermería en la UCAM (Universidad Católica San Antonio de Murcia). Enfermeria Global.

[12] Muller-Botti S, Maestre JM, Del Moral I, Fey M, Simon R (2021). Linguistic validation of the debriefing assessment for simulation in healthcare in spanish and cultural validation for 8 spanish speaking countries. Simulation in Healthcare.

[13] Simon R, Raemer D, Rudolph J (2019). Evaluación del Debriefing para Simulación en Salud (EDSS) Hoja de puntuaciones - Versión del estudiante (extendida). Center of Medical Simulation.

[14] Banerjee A, Slagle JM, Mercaldo ND, Booker R, Miller A, France DJ (2016). A simulation-based curriculum to introduce key teamwork principles to entering medical students. BMC Medical Education.

[15] Rosabel Roig Vila R (Coord.), Antolí Martínez J., Díez Ros R, Pellín Buades N (Eds.) (2020). Memòries del Programa de Xarxes-I3CE de qualitat, innovació i investigació en docència universitària. Convocatòria 2019-20 [Internet].

[16] Leal Costa C, Juguera Rodríguez L, Pardo Ríos M, Martín Roles MR, Díaz Agea JL (2015). Evaluación de cursos de simulación clínica de la Universidad Católica de Murcia (UCAM). Revista Enfermería Docente.

[17] Omer T (2016). Nursing Students’ Perceptions of Satisfaction and Self-Confidence with Clinical Simulation Experience. Journal of Education and Practice.

[18] Durand C, Secheresse T, Leconte M (2017). Intérêt de la grille DASH pour l’évaluation de la qualité des débriefings : étude au cours d’un programme de simulation autour de la réanimation du nouveau-né en salle de naissance. Archives de Pediatrie.

[19] Ciullo A, Yee J, Frey JA, Gothard MD, Benner A, Hammond J (2019). Telepresent mechanical ventilation training versus traditional instruction: A simulation-based pilot study. BMJ Simulation and Technology Enhanced Learning.

[20] Decker S, Alinier G, Crawford SB, Gordon RM, Jenkins D, Wilson C (2021). Healthcare Simulation Standards of Best PracticeTM The Debriefing Process. Clinical Simulation in Nursing.

[21] Hoang C, Copnell B, Lawrence K, Peddle M (2022). Undergraduate Nursing Education and End-of-Life Simulation: A Scoping Review. Clinical Simulation In Nursing.

[22] Brown DK, Kushner Benson SN (2020). Does Time in Team Training Matter? Evaluation of Team-Level Attitudes With Interprofessional Education. Clinical Simulation In Nursing.

[23] Bruce R, Levett-Jones T, Courtney-Pratt H (2019). Transfer of Learning From University-Based Simulation Experiences to Nursing Students’ Future Clinical Practice: An Exploratory Study. Clinical Simulation In Nursing.

[24] Ross JG, Furman G, Scheve A (2024). The Impact of Standardized Patients on First-Year Nursing Students’ Communication Skills. Clinical Simulation In Nursing.

[25] Bozkurt SA, Samia R, Gazarian PK (2023). Using Standardized Patient Simulation in Undergraduate Nursing Education: A Scoping Review. Clinical Simulation In Nursing.

[26] Cole R, Flenady T, Heaton L High Fidelity Simulation Modalities in Preregistration Nurse Education Programs: A Scoping Review. Clinical Simulation In Nursing.

[27] Hémon B, Michinov E, Guy D, Mancheron P, Scipion A (2020). Speaking Up About Errors in Routine Clinical Practice: A Simulation-Based Intervention With Nursing Students. Clinical Simulation In Nursing.

[28] Cant RP, Cooper SJ, Lam LL (2020). Hospital Nurses’ Simulation-Based Education Regarding Patient Safety: A Scoping Review. Clinical Simulation in Nursing.

[29] Klenke-Borgmann L, Mattson N, Peterman M, Stubenrauch C (2023). The Long-Term Transferability of Clinical Judgment Via In-Class Simulations to Nursing Practice: A Qualitative Descriptive Study. Clinical Simulation in Nursing.

[30] El Hussein MT, Cuncannon A (2022). Nursing students’ transfer of learning from simulated clinical experiences into clinical practice: A scoping review. Nurse Education Today.

[31] Alharbi K, Alharbi MF (2022). Nursing Students’ Satisfaction and Self-Confidence Levels After Their Simulation Experience. SAGE Open Nursing.

[32] Hambach C, Cantrell MA, Mariani B (2023). A Program of Simulated Learning Experiences to Develop Clinical Judgment and Clinical Competence Among Sophomore Baccalaureate Nursing Students. Clinical Simulation In Nursing.

